# High (≥6.5) Spontaneous and Persistent Urinary pH Is Protective of Renal Function at Baseline and during Disease Course in Idiopathic Membranous Nephropathy

**DOI:** 10.1155/2015/730234

**Published:** 2015-07-30

**Authors:** Claudio Bazzi, Elena Tagliabue, Sara Raimondi, Virginia Rizza, Daniela Casellato, Masaomi Nangaku

**Affiliations:** ^1^D'Amico Foundation for Renal Disease Research, 20145 Milan, Italy; ^2^Division of Epidemiology and Biostatistics, European Institute of Oncology, 20141 Milan, Italy; ^3^Biochemical Laboratory, San Carlo Borromeo Hospital, 20153 Milan, Italy; ^4^Nephrology and Dialysis Unit, San Carlo Borromeo Hospital, 20153 Milan, Italy; ^5^Division of Nephrology and Endocrinology, University of Tokyo School of Medicine, Tokyo 113-8655, Japan

## Abstract

Metabolic acidosis correction in advanced renal failure slows renal function decline attributed to tubulointerstitial damage (TID) reduction. No study evaluated if spontaneous baseline high urinary pH (UpH) is renoprotective in patients with normal renal function and without metabolic acidosis. The study tested this hypothesis in idiopathic membranous nephropathy (IMN). Eighty-five patients (follow-up 81 ± 54 months) measured UpH, serum creatinine, eGFR, protein/creatinine ratio, fractional excretion of albumin, IgG, *α*1-microglobulin, and urinary N-acetyl-*β*-D-glucosaminidase (*β*-NAG)/creatinine ratio. Twenty-eight patients (33%) had UpH ≥ 6.5 and 57 (67%) pH < 6.5; high versus low UpH patients had significantly lower values of the tubulointerstitial damage (TID) markers FE *α*1m and *β*-NAG and significantly better baseline renal function. These differences persisted over time in a subset of 38 patients with 5 measurements along 53 ± 26 months. In 29 patients with nephrotic syndrome (NS) treated with supportive therapy (follow-up: 80 ± 52 months) renal function was stable in 10 high and significantly worse in 19 low UpH patients. Steroids + cyclophosphamide treatment in 35 NS patients masks the renoprotection of high UpH. *Conclusions*. In IMN high and persistent UpH is associated with reduction of the proteinuric markers of tubulointerstitial damage and baseline better renal function in all patients and in NS patients treated only with supportive therapy during disease course. The factors associated with high pH-dependent renoprotection were lower values of TID markers, eGFR ≥ 60 mL/min, BP < 140/90 mmHg, and age < 55 years.

## 1. Introduction

Metabolic acidosis in CKD is associated with progressive loss of renal function, increased ESRD rate, impaired nutritional parameters, skeletal muscle wasting, bone uremic disease worsening, adverse cardiovascular outcomes, and death [1–5]. Several studies over recent years have evaluated the renoprotective effect of correcting metabolic acidosis (serum bicarbonate < 22 mEq/L) [6] with sodium citrate, sodium bicarbonate, or fruit- and vegetable-rich diet, mainly in patients with advanced renal failure (stage 3-4 CKD) ([7–12]; reviews in [13–18]). These studies showed that the alkalinizing treatment slowed renal function decline, reduced the ESRD rate, ameliorated muscle wasting and nutritional parameters, and reduced the excretion of some urinary markers of kidney injury such as urinary endothelin [8], TGF-*β* [11], angiotensinogen [12], and the tubulointerstitial damage marker N-acetyl-*β*-D-glucosaminidase (*β*-NAG) [8, 9, 11]. The reduced excretion of *β*-NAG associated with reduction of renal function decline observed after 2 years of Na citrate therapy [8], 5 years of NaHCO_3_ therapy [9], and one year of fruit- and vegetable-rich diet [10] suggested that the renoprotective effect of metabolic acidosis correction was dependent on reduction of the extent of tubulointerstitial damage (TID) following alkalinizing treatment. The relationship between metabolic acidosis correction, urinary luminal alkalinization, and reduction of TID has been evaluated in several studies. Cell culture and remnant kidney models [19, 20] showed that tubular protein-overload activates complement at the brush border of proximal tubular epithelial cells (PTECs), inducing tubular damage and interstitial inflammatory cells infiltration. These observations were confirmed in experimental models of MN that showed the role of complement activation in PTECs as responsible of tubulointerstitial damage. The mechanisms of complement activation in PTECs have been evaluated in some studies. In a mouse model of protein-overload nephropathy urine luminal alkalinization induced by NaHCO_3_ feeding attenuates the proteinuria-induced oxidative damage in PTECs. Couser and Nangaku [21] suggested that the protective effect of NaHCO_3_ is dependent on reduced intratubular complement activation, as shown in some experimental models [22, 23]. Moreover a cell culture study [24] showed that the deposition of C3 and C9 on the surface of HK-2 cells is maximal at acidic pH values and significantly reduced at pH ≥ 6.5. A study of patients with glomerular diseases [25] showed that the urinary excretion of complement activation products increased significantly with higher levels of proteinuria in all diseases except minimal change disease and decreased significantly after two weeks of sodium bicarbonate administration. As a whole the results of these studies in experimental models and human diseases suggest that the tubulointerstitial damage is dependent at least on two factors: the tubular load of proteins and the activation of complement in tubular cells whose level is reduced by luminal alkalinization. The studies on metabolic acidosis correction in advanced renal failure showed that the reduction of renal function decline was associated with a reduced excretion of the tubular damage marker *β*-NAG. The overall conclusion of the studies on metabolic acidosis correction in advanced renal failure is that this treatment is an inexpensive and simple therapeutic strategy with an effective kidney protective value adjunct to blood pressure control with angiotensin-converting enzyme inhibition. The published studies were limited by the inclusion almost exclusively of patients with advanced renal failure (stage 3-4 CKD) and lack of diagnosis in most of them except for hypertensive nephropathy in 2 studies; only one study showed greater improvement in patients with eGFR > 45 mL/min/1.73 m^2^. If the renoprotective mechanism does in fact stem from a reduction of tubular damage mediated by reduced complement activation consequent to luminal alkalinization, it would be interesting to evaluate whether high spontaneous and persistent urinary pH is renoprotective not only in patients with advanced renal failure and metabolic acidosis but also in patients with normal renal function without metabolic acidosis. To test this hypothesis we evaluated renal function parameters and some proteinuric markers of tubulointerstitial damage in 85 patients with idiopathic membranous nephropathy (IMN), mainly with normal renal function at biopsy (68% with eGFR ≥ 60 mL/min/1.73 m^2^) and a rather long follow-up (81 ± 54 months) to assess whether spontaneous baseline (≥6.5) and persistent high urinary pH is renoprotective in terms of reduced tubulointerstitial damage, better baseline renal function, and slower renal function decline during the disease course. A second aim was to assess whether chronic renal failure (eGFR < 60 mL/min/1.73 m^2^), age, and high blood pressure may interfere with the renoprotective effects of spontaneous high pH.

## 2. Patients

Eighty-five patients with IMN were included in the study; the patients were part of a cohort of 105 IMN patients diagnosed between 1992 and 2005 in the Nephrology and Dialysis Unit of the San Carlo Borromeo Hospital. Inclusion criteria were as follows: typical features of IMN at light and immunofluorescence microscopy; no clinical and/or laboratory signs of secondary MN; clinical presentation with nephrotic syndrome (NS: 82%) or persistent nonnephrotic proteinuria (PP: 18%); at least 6 glomeruli in renal biopsy; follow-up of at least 12 months. Exclusion criteria were as follows: lack of follow-up (*N* 18); NS with haemodynamic acute reversible renal failure at biopsy (*N* 2). The baseline clinical and laboratory characteristics of all patients are reported in Table 1. Twenty-nine patients with NS were treated only with supportive therapy: diuretics, antihypertensives (including ACEi/ARBs), statins, antiplatelet agents, and vitamin D3 when indicated. Thirty-five NS patients, besides supportive therapy, were treated soon after diagnosis with steroids and cyclophosphamide for six months according to the Ponticelli protocol [26]. Six NS patients were treated with steroids alone: prednisone 1 mg/kg/day with tapering for 4–12 months. Fifteen patients with PP were treated only with supportive therapy. The patients were screened up until the last planned follow-up clinic visit in 2011; the overall follow-up was 81 ± 54 months (range 12–226). The primary outcome was the level of renal function parameters at baseline and during disease course; a secondary outcome was progression to ESRD.

## 3. Analytical Methods

Renal biopsies were performed and evaluated by the previously described standard histological and immunofluorescence methods [27]. Total urinary proteins were measured by the Coomassie blue method in second morning urine sample and expressed as protein/creatinine ratio (P/C: mg of proteins/1 g urinary creatinine). Serum (sCr) and urinary creatinine were measured automatically on a Beckman LX20 analyzer using the Jaffè method and expressed in mg/dL. Estimated Glomerular Filtration Rate (eGFR) was calculated according to the 4-variable MDRD formula [28]; the body weight in high and low UpH groups was 62 ± 12 kg (46–83) and 63 ± 9 kg (45–85), respectively. *β*-NAG was measured in the second morning urine sample by a colorimetric method as described [29] and expressed as the *β*-NAG/creatinine ratio divided by the eGFR (NAG/C/eGFR) [30], as *β*-NAG excretion is dependent on the functioning nephron mass; this method of calculation was suggested by Ellam and El Nahas [31] which observed that the urinary excretion of proteins should be adjusted for the functioning nephron mass of whom eGFR may be a reliable index. All patients were measured at biopsy for several urinary proteins of different MW: IgG (MW 150 kDa), albumin (Alb, MW 67 kDa), and *α*1-microglobulin (*α*1m : MW 31.8 kDa). The proteins were measured by BNA nephelometer (Behring, Milan, Italy) using rabbit serum antibodies (Behring). The excretion of albumin, IgG, and *α*1m was expressed as fractional excretion (FE) according to the formula: [(urinary protein/serum protein × sCr/uCr) × 100]. Urinary pH was measured by Multistix (Siemens) soon after urine collection.

## 4. Statistical Methods

Statistical analysis was performed using SAS 9.2. Patients were divided into two groups based on the second tertile of basal UpH distribution, that is, 6.5. Differences in baseline characteristics between high and low UpH groups were determined using the Mann-Whitney test and the chi-square test for continuous and categorical variables, respectively. Differences in the high and low UpH groups between baseline and last observed creatinine and eGFR values were calculated by the Wilcoxon signed-ranks test for repeated measures. Correlations were assessed using the Spearman rank test. Differences in ESRD rate were evaluated using Kaplan-Meier survival curves; the equality of survival curves was assessed by log-rank test. Multivariate Cox proportional hazard regression analysis was performed on the population as a whole. Statistical significance was defined as *p* < 0.05.

## 5. Results

### 5.1. Baseline and Follow-Up pH

At baseline 28 patients (33%) had urinary pH ≥ 6.5 (high pH group: “high UpH”) and 57 patients (67%) had pH < 6.5 (low pH group: “low UpH”) (Table 1). The baseline pH in high versus low UpH group was 6.91 ± 0.36 and 5.50 ± 0.47, respectively (*p* < 0.0001). Serum bicarbonate was not significantly different between the high and low UpH groups. The percentage of patients with eGFR <60 mL/min/1.73 m^2^ was not significantly different between the high and low UpH groups (25% versus 32%, *p* = 0.35). Twenty-three patients in the high UpH group and 43 patients in the low UpH group had several measurements of pH, functional, and proteinuric parameters (on average 5 measurements over time from 6 to 121 months). The baseline values of all functional and proteinuric parameters of patients included or not included in the follow-up study were not significantly different (data not shown). In patients with baseline pH ≥ 6.5 the mean follow-up UpH was significantly higher than in patients with baseline pH < 6.5 (6.45 ± 0.50 versus 5.65 ± 0.40, resp., *p* < 0.0001). Correlation analysis showed that none of the clinical, functional, and proteinuric parameters was correlated with the pH value; it is reasonable to suppose that the different pH levels may be at least partly related to different dietary habits; unfortunately no information is available about the dietary habits of the patients.

### 5.2. Comparison of Baseline Clinical, Functional, and Proteinuric Parameters in High versus Low UpH Patients

In patients with high versus low UpH (Table 1), at baseline the markers of tubulointerstitial damage FE *α*1m (*p* = 0.02) and *β*-NAG/C/eGFR (*p* = 0.04) were significantly lower; the renal function parameters sCr and eGFR were significantly better (sCr: 0.99 ± 0.34 versus 1.31 ± 0.57 mg/dL, *p* = 0.003; eGFR 83 ± 28 versus 68 ± 27, *p* = 0.03). Conversely the proteinuric markers for altered glomerular filtration barrier (P/C ratio, FE albumin, and FE IgG) were not significantly different between the two groups. Limiting the analysis to patients with eGFR ≥ 60 mL/min/1.73 m^2^ in 21 high UpH versus 37 low UpH sCr was significantly lower (*p* = 0.004), eGFR higher at the limit of significance (*p* = 0.05), and FE *α*1m significantly lower (*p* = 0.03). Thus spontaneous high UpH was significantly associated at baseline with better renal function and lower values of the proteinuric markers for TID.

### 5.3. Comparison of Baseline Functional and Proteinuric Parameters between High versus Low UpH Groups in the Subset of 70 Patients with Nephrotic Syndrome

Limiting the analysis to 70 patients with NS in the high UpH (number 25) versus low UpH group (number 45), the sCr was significantly lower (*p* = 0.008) and eGFR higher at the limit of statistical significance (*p* = 0.050); P/C ratio, FE albumin, and FE IgG were not significantly different, while the TID markers FE *α*1m (*p* = 0.01) and *β*-NAG/C/GFR (*p* = 0.009) were significantly lower (data not shown).

### 5.4. Comparison of Functional and Proteinuric Parameters at Baseline and at the Last Measurement in a Subset of 38 Patients (11 High UpH, 27 Low UpH) with 5 Serial Measurements along 56 ± 26 Months (Range 24–121)

To assess whether the differences between the high and low UpH group observed at baseline were persistent over time, of the 66 patients with several measurements, a subgroup of 38 patients was selected who had 5 serial measurements of all parameters at least 24 months after baseline (follow-up: 53 ± 26 months; range: 24–121): 11 patients of the high and 27 of the low UpH group. The mean urinary pH during this time was significantly higher in high versus the low UpH group (6.32 ± 0.37 versus 5.66 ± 0.43, *p* = 0.0001). In the high UpH group (follow-up: 57 ± 30 months) the last values of the proteinuric markers of TID and the functional parameters sCr and eGFR were not significantly different from the baseline values (Table 2). Conversely, in the low UpH group (follow-up: 51 ± 24 months), at the last measurement, sCr was significantly higher (*p* = 0.001), eGFR significantly lower (<0.0001), and FE *α*1m significantly higher (*p* = 0.01), while *β*-NAG/C/eGFR was not significantly different. Thus serial measurements of functional and proteinuric parameters during a rather long follow-up confirm that at the last observation spontaneous and persistent high UpH is associated with stable renal function, while low UpH is associated with worse renal function and higher levels of FE *α*1m.

### 5.5. Renoprotective Value of Spontaneous Baseline and Persistent High UpH over Time in 29 NS Patients Treated Only with Supportive Therapy

The renoprotective value of spontaneous high UpH was evaluated in 29 NS patients treated only with supportive therapy (10 with high UpH, 19 with low UpH patients). At baseline, the proteinuric TID markers were significantly lower (FE *α*1m, *p* = 0.01; *β*-NAG/C/eGFR, *p* = 0.04), sCr was significantly lower (*p* = 0.013), and eGFR was significantly higher (*p* = 0.007) in high versus low UpH patients (Table 3); none of the “glomerular” markers (P/C ratio, FE albumin, and FE IgG) was significantly different between the two groups. At the last observation after a follow-up of 86 ± 55 months, in the high UpH group sCr and eGFR levels were not significantly different from the baseline values (sCr: 0.83 ± 0.12 versus 1.05 ± 0.40, *p* = 0.11; eGFR 97 ± 19 versus 78 ± 29, *p* = 0.12). In the low UpH group, at the last observation after a follow-up of 75 ± 52 months, sCr and eGFR were significantly worse (sCr: 3.30 ± 2.75 versus 1.18 ± 0.39, *p* = 0.027, eGFR: 44 ± 39 versus 69 ± 27, *p* = 0.026), and the TID markers were significantly higher: FE *α*1m 0.480 ± 0.440 versus 0.290 ± 0.340, *p* = 0.02; *β*-NAG/C/eGFR 0.35 ± 0.32 versus 0.21 ± 0.23, *p* = 0.04. Progression to ESRD was higher in low versus high UpH patients (37% versus 0%) but the difference did not attain statistical significance (*p* = 0.12). Thus the patients in the high UpH group treated with supportive therapy have not only significantly better baseline functional parameters and lower TID markers but also stable renal function over time. By contrast, the patients of the low UpH group had worse renal function and increased TID markers both at baseline and during disease course.

### 5.6. Renal Function over Time in 35 NS Patients Treated with Steroids and Cyclophosphamide

Thirty-five NS patients were treated with steroids and cyclophosphamide according to the Ponticelli protocol [26] (15 with high and 20 with low UpH). At the last observation the sCr and eGFR values of the high UpH group (follow-up: 76 ± 47 months) were improved but not significantly versus baseline values (sCr: 1.16 ± 0.38 versus 2.34 ± 2.50 mg/dL, *p* = 0.09; eGFR: 68 ± 26 versus 61 ± 38 mL/min/1.73 m^2^, *p* = 0.54). Also in the low UpH group (follow-up: 112 ± 63 months), at the last observation, sCr and eGFR were not significantly improved versus baseline values (sCr: 1.38 ± 0.63 versus 1.93 ± 1.76 mg/dL, *p* = 0.20; eGFR: 67 ± 24 versus 59 ± 31 mL/min/1.73 m^2^, *p* = 0.37). These results suggest that immunosuppressive therapy may mask the renoprotective effect of spontaneous high urinary pH.

### 5.7. Factors Affecting the Renoprotective Effect of High Urinary pH

Three parameters were considered for their possible influence on renal function and proteinuric markers in high versus low UpH patients: age below or above the median value (< versus ≥55 years), eGFR (≥ versus <60 mL/min/1.73 m^2^), and baseline blood pressure (< versus ≥140/90 mmHg). In patients with high versus low UpH and age < 55 years, at baseline sCr was significantly lower (*p* = 0.002) and eGFR was significantly higher (*p* = 0.004); P/C ratio, FE Alb, and FE IgG were not significantly different; FE *α*1m was significantly lower (*p* = 0.02) and *β*-NAG/C/eGFR was lower at the limit of statistical significance (*p* = 0.05). In patients aged ≥55 years none of the parameters was significantly different between high and low UpH. In patients with eGFR ≥ 60 mL/min/1.73 m^2^ and high versus low UpH, at baseline sCr was lower (*p* = 0.004) and eGFR was higher at the limit of statistical significance (*p* = 0.05); none of the “glomerular” proteinuric parameters were significantly different; the TID marker FE *α*1m was significantly lower (*p* = 0.03) while *β*-NAG/C/eGFR was not significantly different (*p* = 0.40). In patients with eGFR < 60 mL/min/1.73 m^2^ none of the parameters was significantly different between the high and low UpH groups. In patients with baseline BP < 140/90 mmHg sCr (*p* = 0.006), FE *α*1m (*p* = 0.01), and *β*-NAG/C/eGFR (*p* = 0.02) were significantly lower in high versus low UpH group; eGFR was at the limit of significance (*p* = 0.07). In patients with BP ≥ 140/90 mmHg all parameters were not significantly different between UpH groups. This data suggests that spontaneous high pH is associated with better renal function mainly in patients with age < 55 years, eGFR ≥ 60 mL/min/1.73 m^2^, and normal BP; the proteinuric markers for TID were significantly lower in patients with age < 55 years and normal BP, but not in patients with eGFR ≥ 60 mL/min/1.73 m^2^.

### 5.8. Multivariate Cox Regression Analysis

The results of multivariate Cox regression analysis including age, eGFR, fractional excretion of *α*1m, fractional excretion of IgG, and pH showed that none of the markers was significantly associated with progression.

## 6. Discussion

The majority of studies showing a reduction in renal function decline following treatment of metabolic acidosis with alkalinizing drugs or foods were performed in patients with advanced renal failure (stages 3-4). Our study evaluated for the first time in a cohort of IMN patients whether high spontaneous baseline and persistent urinary pH may be renoprotective both at baseline and during the disease course in patients without metabolic acidosis. The study was also aimed at assessing which factors might be associated with the renoprotective effect of high UpH. In our cohort, 28 patients had baseline urinary pH ≥ 6.5 significantly higher than in 57 patients with baseline urinary pH < 6.5. In the high UpH group the values of pH remained significantly higher on average in several measurements performed over a mean period of about 5 years compared to patients with low UpH. At baseline, high UpH was associated with significantly lower values of the proteinuric TID markers FE *α*1m and *β*-NAG/C/eGFR and better renal function in comparison with patients with low UpH. In patients treated only with supportive therapy, those with high UpH had stable renal function over a period of about 7 years: by contrast patients with low UpH were characterized by significantly worse renal function and significantly higher levels of the TID markers over a period of about 6 years. The ESRD rate was higher in low versus high UpH patients (37% versus 0%) but the difference did not attain a statistical significance (*p* = 0.12). Renal function worsening in low UpH patients cannot be attributed to higher levels of the “glomerular” proteinuric markers for altered filtration barrier (P/C ratio, FE albumin, and FE IgG) known to be associated with progressive renal disease as these markers were not significantly different between low versus high UpH patients. Conversely the significant reduction in high UpH patients of the proteinuric markers of tubulointerstitial damage FE *α*1m and *β*-NAG may be possibly dependent on lower complement activation consequent to urinary alkalinization as observed in some experimental models and clinical studies. One functional (eGFR) and two clinical (age, blood pressure) parameters were evaluated as factors that might possibly influence the renoprotective effect of spontaneous high pH: the results showed that, in patients with eGFR ≥ 60 mL/min/1.73 m^2^, age < 55 years, and BP < 140/90 mmHg, the baseline functional parameters were significantly better; also the proteinuric TID markers were significantly lower in high versus low UpH, except in patients with eGFR ≥ 60 mL/min/1.73 m^2^. By contrast, in patients with eGFR ≤60 mL/min/1.73 m^2^, age ≥ 55 years, and BP ≥ 140/90 mmHg both the functional parameters and proteinuric TID markers were not significantly different between high and low UpH. These data suggest that four factors are associated with better renal function in IMN patients with spontaneous and persistently high urinary pH: a less severe tubulointerstitial damage, eGFR ≥ 60 mL/min/1.73 m^2^, age < 55 years, and BP < 140/90 mmHg. This observation could suggest that spontaneous high UpH is less powerful in reducing renal function decline and TID markers than the metabolic acidosis correction that reduces renal function decline and TID markers also in patients with advanced renal failure (stages 3-4). The renoprotective effect of spontaneous high urinary pH in IMN patients is a new rather unexpected and interesting observation. If confirmed in prospective controlled trials, the main clinical message could be that, in IMN patients with normal renal function, those with low urinary pH could usefully be given alkalinizing drugs as a cheap and simple treatment strategy, with an effective kidney protective value adjunct to blood pressure control with angiotensin-converting enzyme inhibition. None of our patients with advanced renal failure (stages 3-4) was treated with alkalinizing therapy; obviously our observations do not rule out the possibility that also in IMN patients with advanced renal failure the metabolic acidosis correction with NaHCO_3_, Na citrate, or alkalinizing foods may be renoprotective.

## 7. Conclusions

The study shows for the first time that in IMN the patients with high spontaneous and persistent urinary pH (≥6.5) in comparison with patients with lower pH (<6.5) have significantly better renal function at baseline and during disease course in patients treated only with supportive therapy. The main factor associated with high pH-dependent renoprotection is the significant reduction of the tubulointerstitial damage markers FE *α*1m and *β*-NAG, possibly dependent on less complement activation in tubular cells due to urinary alkalinization as observed in some experimental and clinical studies.

## Figures and Tables

**Table 1:** Comparison of baseline and follow-up urinary pH and clinical, functional, and proteinuric parameters in 85 IMN patients with pH ≥ 6.5 (*N* 28) or pH < 6.5 (*N* 57).

Parameter	All patients	Pts. with pH ≥ 6.5	Pts. with pH < 6.5	*p* value
	(*N* = 85)	(*N* = 28)	(*N* = 57)	
	Mean (±SD)	Mean (±SD)	Mean (±SD)	
Baseline pH	5.96 ± 0.79	6.91 ± 0.36	5.50 ± 0.47	**<0.0001**
Follow-up pH	5.92 ± 0.58 (*N* 66)	6.44 ± 0.47 (*N* 23)	5.65 ± 0.47 (*N* 43)	**<0.0001**
Serum bicarbonate mEq/L	26.8 ± 3.4	27.7 ± 3.8	26.4 ± 3.6	0.13
Age (years)	52 ± 17	52 ± 21	52 ± 15	0.95
sCr (mg/dL)	1.20 ± 0.53	0.99 ± 0.34	1.31 ± 0.57	**0.003**
eGFR (mL/min/1.73 m^2^)	73 ± 28	83 ± 28	68 ± 27	**0.03**
eGFR < 60 mL/min/1.73 m^2^ (%)	32%	25%	35%	0.35
BP ≥140/90 mmHg (%)	53%	36%	61%	**0.03**
P/C ratio	3731 ± 2573	3649 ± 2288	3772 ± 2721	0.99
FE albumin	0.180 ± 0.170	0.160 ± 0.150	0.190 ± 0.180	0.50
FE IgG	0.05 ± 0.06	0.04 ± 0.06	0.05 ± 0.07	0.27
FE *α*1m-microglobulin	0.420 ± 0.410	0.290 ± 0.340	0.480 ± 0.440	**0.02**
NAG/C/eGFR	0.30 ± 0.30	0.21 ± 0.23	0.35 ± 0.32	**0.04**

Note: significant *p* values are in bold.

**Table 2:** Comparison of functional and proteinuric parameters at baseline and at the last observation in a subset of 38 IMN patients (high UpH group *N* 11, low UpH group *N* 27) with 5 serial measurements along 56 ± 26 months (range 24–121).

Parameter	UpH ≥ 6.5 (*N* = 11)		*p* value	UpH < 6.5 (*N* = 27)		*p* value
	Baseline values	Last values		Baseline values	Last values	
	Mean (±SD)	Mean (±SD)		Mean (±SD)	Mean (±SD)	
Follow-up (months)		57 ± 30			51 ± 25	0.57
Mean follow-up pH		6.32 ± 0.37			5.66 ± 0.43	**0.0001**
sCr (mg/dL)	0.89 ± 0.33	1.23 ± 1.36	0.46	1.27 ± 0.54	2.43 ± 2.13	**0.001**
eGFR mL/min/1.73 m^2^	94 ± 32	91 ± 40	0.81	69 ± 27	51 ± 34	**<0.0001**
P/C ratio	2867 ± 2117	2091 ± 3122	0.37	4399 ± 3115	3881 ± 4350	0.43
FE albumin	0.106 ± 0.144	0.165 ± 0.328	0.65	0.179 ± 0.137	0.315 ± 0.454	0.35
FE IgG	0.029 ± 0.064	0.062 ± 0.163	0.97	0.059 ± 0.067	0.150 ± 0.280	0.34
FE *α*1m	0.195 ± 0.212	0.503 ± 1.183	0.97	0.476 ± 0.495	1.663 ± 2.300	**0.01**
NAG/C/eGFR	0.157 ± 0.228	0.416 ± 0.936	0.70	0.342 ± 0.328	0.791 ± 1.088	0.23
ACE-inhibitors treatment (%)		36%			59%	0.17
Steroid + cyclophosphamide treatment (%)		36%			37%	n.s.

Note: significant *p* values are in bold.

**Table 3:** Baseline and last observation clinical, functional, and proteinuric parameters in 29 IMN with NS patients treated with supportive therapy with UpH ≥ 6.5 (*N* 10) or UpH < 6.5 (*N* 19).

Parameter	Patients with pH ≥ 6.5 (*N* = 10)	Patients with pH < 6.5 (*N* = 19)	*p* value
	Mean (±SD)	Mean (±SD)	
Baseline pH	7.00 ± 0.33	5.56 ± 0.47	**<0.0001**
Follow-up pH	6.36 ± 0.43	5.75 ± 0.37	**0.005**
BP ≥140/90 mmHg (%)	10%	74%	**0.002**
Baseline sCr (mg/dL)	0.83 ± 0.12^∧^	1.18 ± 0.39^§^	**0.013**
Last observation sCr (mg/dL)	1.05 ± 0.40^∧^	3.30 ± 2.70^§^	**0.027**
Baseline eGFR (mL/min/1.73 m^2^)	97 ± 19^*∗*^	69 ± 26°	**0.007**
Last observation eGFR (mL/min/1.73 m^2^)	78 ± 30^*∗*^	44 ± 39°	**0.026**
ESRD (%)	0%	37%	0.12
Follow-up (months)	86 ± 55	75 ± 52	0.49
P/C ratio	3570 ± 1996	4808 ± 3361	0.41
FE Alb	0.103 ± 0.056	0.201 ± 0.150	0.11
FE IgG	0.013 ± 0.011	0.062 ± 0.070	0.07
FE *α*1m	0.170 ± 0.110	0.499 ± 0.440	**0.01**
NAG/C/eGFR1	0.134 ± 0.06	0.434 ± 0.400	**0.04**

Note: significant *p* values are in bold.

^∧^ *p* = 0.12; ^§^ **p** = 0.003; ^*∗*^ *p* = 0.11; °**p** = 0.02.
